# Vitamin K supplementation and vascular calcification: a systematic review and meta-analysis of randomized controlled trials

**DOI:** 10.3389/fnut.2023.1115069

**Published:** 2023-05-12

**Authors:** Te Li, Yun Wang, Wei-ping Tu

**Affiliations:** Department of Nephrology, The Second Affiliated Hospital of Nanchang University, Nanchang, Jiangxi, China

**Keywords:** vitamin K, vascular calcification, coronary artery calcification, dephospho-uncarboxylated matrix Gla protein, systematic review, meta-analysis, randomized controlled trials

## Abstract

**Background:**

Vascular calcification (VC) is a complex process that has been linked to conditions including cardiovascular diseases and chronic kidney disease. There is an ongoing debate about whether vitamin K (VK) can effectively prevent VC. To assess the efficiency and safety of VK supplementation in the therapies of VC, we performed a systematic review and meta-analysis of recent studies.

**Methods:**

We searched major databases, including PubMed, the Cochrane Library, Embase databases, and Web of Science up until August 2022. 14 randomized controlled trials (RCTs) describing the outcomes of treatment for VK supplementation with VC have been included out of 332 studies. The results were reported in the change of coronary artery calcification (CAC) scores, other artery and valve calcification, vascular stiffness, and dephospho-uncarboxylated matrix Gla protein (dp-ucMGP). The reports of severe adverse events were recorded and analyzed.

**Results:**

We reviewed 14 RCTs, comprising a total of 1,533 patients. Our analysis revealed that VK supplementation has a significant effect on CAC scores, slowing down the progression of CAC [*I*^2^ = 34%, MD= −17.37, 95% CI (−34.18, −0.56), *p* = 0.04]. The study found that VK supplementation had a significant impact on dp-ucMGP levels, as compared to the control group, where those receiving VK supplementation had lower values [*I*^2^ = 71%, MD = −243.31, 95% CI (−366.08, −120.53), *p* = 0.0001]. Additionally, there was no significant difference in the adverse events between the groups [*I*^2^ = 31%, RR = 0.92, 95% CI (−0.79,1.07), *p* = 0.29].

**Conclusion:**

VK may have therapeutic potential for alleviating VC, especially CAC. However, more rigorously designed RCTs are required to verify the benefits and efficacy of VK therapy in VC.

## Introduction

Vascular calcification (VC) is an independent predictor of morbidity and mortality in a variety of diseases, including cardiovascular disease (CVD) and chronic kidney disease (CKD) (1). The ongoing progression of VC causes impaired compliance of vascular, atherosclerotic plaque rupture, and thrombosis. Furthermore, VC occurrences in young individuals have also sharply increased in the context of diabetes mellitus (DM), CKD, and atherosclerosis (2). Based on the available evidence, it is crucial to recognize the role of VC in the development of vascular damage. However, the creation of efficient prevention and drug therapeutic methods continues to be a serious clinical challenge due to the existing inadequate knowledge about VC.

Vitamin K (VK) is a fat-soluble vitamin with three different forms as follows: VK1 (phylloquinone), VK2 (menaquinone), and VK3 (menadione) (3). VK1 is the main source in Western countries, mainly found in green vegetables (such as spinach and broccoli) and vegetable oils (such as soybeans, olive oil, and canola). VK2, produced by gut bacteria, is less common in the diet and is found in fermented foods, such as cheese and meat. VK3 is a synthetic form that is often added to animal feed (4). VK has several crucial functions in the body. VK1 plays the most important role in the activation of blood clotting factors in the liver, while VK2 plays a role in protein synthesis in extrahepatic tissues such as blood vessel walls (5). In recent years, the awareness of the role of VK has grown significantly because of its well-known involvement in several diseases, such as osteoporosis (6), CVD (7), inflammation (8), cancer (9), Alzheimer's disease (10), and peripheral neuropathy (11). A growing body of research indicates that VK has a positive impact on cardiovascular health, providing a low-cost and safe treatment option (12, 13).

VC is a chronic inflammatory process that involves the activation of macrophages and the differentiation of vascular smooth muscle cells into osteoblasts within the intimal and medial layers of artery walls. This process is facilitated by the generation of pro-inflammatory cytokines, such as interleukin-1 (IL-1), IL-6, and tumor necrosis factor α (TNF-α). This process could be primarily mediated by the NF-κB pathway (14). Previous studies have suggested that VK exhibits anti-inflammatory properties by inhibiting the NF-κB pathway, as demonstrated in both *in vitro* and *in vivo* studies. These findings suggest that VK supplementation may help inhibit VC through anti-inflammatory mechanisms (15). Moreover, VK is necessary for the activation of several proteins involved in VC. These vitamin K-dependent proteins (VKDPs) include matrix Gla proteins (MGP), growth arrest-specific 6 (Gas 6), and Gla-rich protein (GRP). MGP is an important tissue calcification inhibitor that helps to prevent both intimal and medial VC. VK may also slow the progression of VC by increasing MGP activity by facilitating its carboxylation (16). Similarly, the inhibitory activity of GRP calcification relies on the post-translational carboxylation of Glu residues. The undercarboxylated protein lacks calcification inhibitory capacity, as demonstrated by its potential to act as an inhibitor of VC (17). Gla-rich protein is a ligand for the Tyro3/Axl/Mer (TAM) family of receptor tyrosine kinases that inhibits VC by preventing endothelial cell and vascular smooth muscle cells from going through apoptosis (18). These VKDPs are thought to carry out their intended functions through γ-carboxylation with VK, and different VKDPs may have synergistic or antagonistic effects on each other (19).

Overall, VK supplementation may provide a simple and relatively safe therapeutic strategy for preventing the development of VC, particularly in individuals who are at high risk of CVD and are prone to VK deficiency. Previous studies have produced conflicting results on the effectiveness of VK supplementation in improving measures of VC, with some studies reporting positive effects (12, 13) and others reporting no improvement (20, 21). Based on this, we performed an updated systematic review and meta-analysis of randomized controlled trials (RCTs) to confirm the relationship between VK supplementation and VC diseases.

## Methods

### Literature search

This article was reported in accordance with the Preferred Reporting Items for Systematic Reviews and Meta-Analysis (PRISMA) statement (22). From the period of their creation until August 2022, PubMed, Web of Science, the Cochrane Library, and Embase databases were used in our literature searches. The following search phrases were used: “vascular calcification or Calcification, Vascular or Calcifications, Vascular or Vascular Calcifications or Vascular Calcinosis or Calcinoses, Vascular or Calcinosis, Vascular or Vascular Calcinoses” and “Vitamin k1 or Phytonadione or Vitamin K1 or Phytomenadione or Phylloquinone or Phyllohydroquinone or Aquamephyton or Konakion or Vitamin k2 or Menaquinones or Vitamin K2 or Menaquinone or Vitamin K Quinone” and “Randomized Controlled Trial or random^*^.” For more details regarding the search strategy used for each database, please refer to Supplementary Document 1. According to PICOS criteria to develop a retrieval strategy summarized in Table 1.

**Table 1:** Population, intervention, comparison, outcomes, and settings (PICOS) criteria for the inclusion of studies evaluating the effects and safety of VK intake on vascular properties.

**Parameter**	**Inclusion criteria**
P (population)	Adults (year ≥18)
I (intervention)	VK (VK1 or VK2) supplementation
C (COMPARISON)	Non-exposed control group
O (outcomes)	Any measurement of VC, dp-ucMGP, adverse advents
S (settings)	RCTs

### Study selection criteria and study types

#### Inclusion criteria

Articles available in English reporting RCTs involving adult participants (age ≥18 years) of any race. These articles must have concerned VK supplementation with a placebo or no-treatment control group, and the report must include an indicator associated with VC at the baseline and study endpoint. Co-interventions were regarded as acceptable as long as both groups received them.

#### Exclusion criteria

Other cohort clinical trials or observational studies. Case reports, reviews, comments, letters, animal studies, and studies containing mixed pediatric and adult populations. Literature that cannot provide relevant raw data was also excluded.

### Evaluation outcomes

The primary outcome was artery calcification: the change in CAC scores, artery volume, and others. We identified the following as suitable methods for evaluating VC: ① plain lateral abdominal X-ray, ② computed tomography (CT) measuring vessels and valve calcification, volume or mass calcification scores, and ③ ^18^F-Sodium Fluoride Positron Emission Tomography (^18^F-NaF PET) imaging. The secondary outcomes were dp-ucMGP and adverse events.

### Study selection and data extraction

Two unbiased reviewers Te Li and Yun Wang used a study selection sheet to assess the articles acquired through the search strategy. Studies that did not fit the inclusion criteria were excluded after the titles and abstracts were initially screened. After a preliminary examination, the studies that had not been excluded were retrieved for full-text screening. It was decided whether the study should be considered for inclusion in our analysis based on the inclusion criteria. During the whole process, if there is a dispute, the final decision for inclusion was made by consensus by Weiping Tu.

The data were extracted by two unbiased reviewers Te Li and Yun Wang, including study characteristics (authors, publication year, country, and the number of centers), study participants (such as eligibility criteria and baseline characteristics), and study design traits. Others including study interventions, controls, period of treatment, length of follow-up, and study results were also extracted.

### Bias, quality, sensitivity analysis, and subgroup analysis assessments

The intervention quality of the studies was evaluated for the risk of bias using the Cochrane Risk of Bias tool for RCTs (23). The risk was estimated using the six criteria listed below: adequate sequence generation, allocation concealment, blinding, incomplete data outcome, and selective reporting. According to these criteria, the risk was classified as low high, or unclear risk of bias. Sensitivity analyses and subgroup analyses were conducted to minimize inter-study heterogeneity. Sensitivity analyses were performed by eliminating one study at a time. Based on participants (hemodialysis vs. no-hemodialysis) and the date of publication (before 2015 vs. after 2015), subgroup analyses were undertaken.

### Statistical analysis

Our article was produced utilizing the Review Manager version 5.4, Stata version 17, and SPSS version 26.0 (IBM Corp, Armonk, NY, United States) for analysis. For dichotomous or polytomous outcomes (adverse events), risk ratios (RRs) were calculated. The use of mean differences (MDs) and 95% confidence intervals (CIs) was evaluated for continuous variables. From the treatment and control groups, MD and standard deviation (SD) for VC and dp-ucMGP were extracted. If MD and SD for outcome indicators of interest were not reported, other available data were used for calculation. The MD and SD in VC were calculated from the median, interquartile range, and sample size. SD of VC or dp-ucMGP was calculated using standard methods based on the mean and 95% CIs or mean and *p*-value. Cochrane's *Q*-test and *I*^2^ statistic were applied to examine the statistical heterogeneity (≥75%, high heterogeneity; 51%−75%, moderate heterogeneity; 26%−50%, low heterogeneity; and ≤25%, insignificant heterogeneity). It is worth noting that the *I*^2^ estimates with 95% CIs can be used to assess heterogeneity, but it is possibly associated with fluctuation in meta-analysis with <15 trials (24). For the data analysis, we employed a random effect model if there was significant heterogeneity. If there was no significant heterogeneity, we chose a fixed effect model. Statistical significance was defined as a *p*-value of <0.05.

## Results

### Study selection

**Figure 1:**
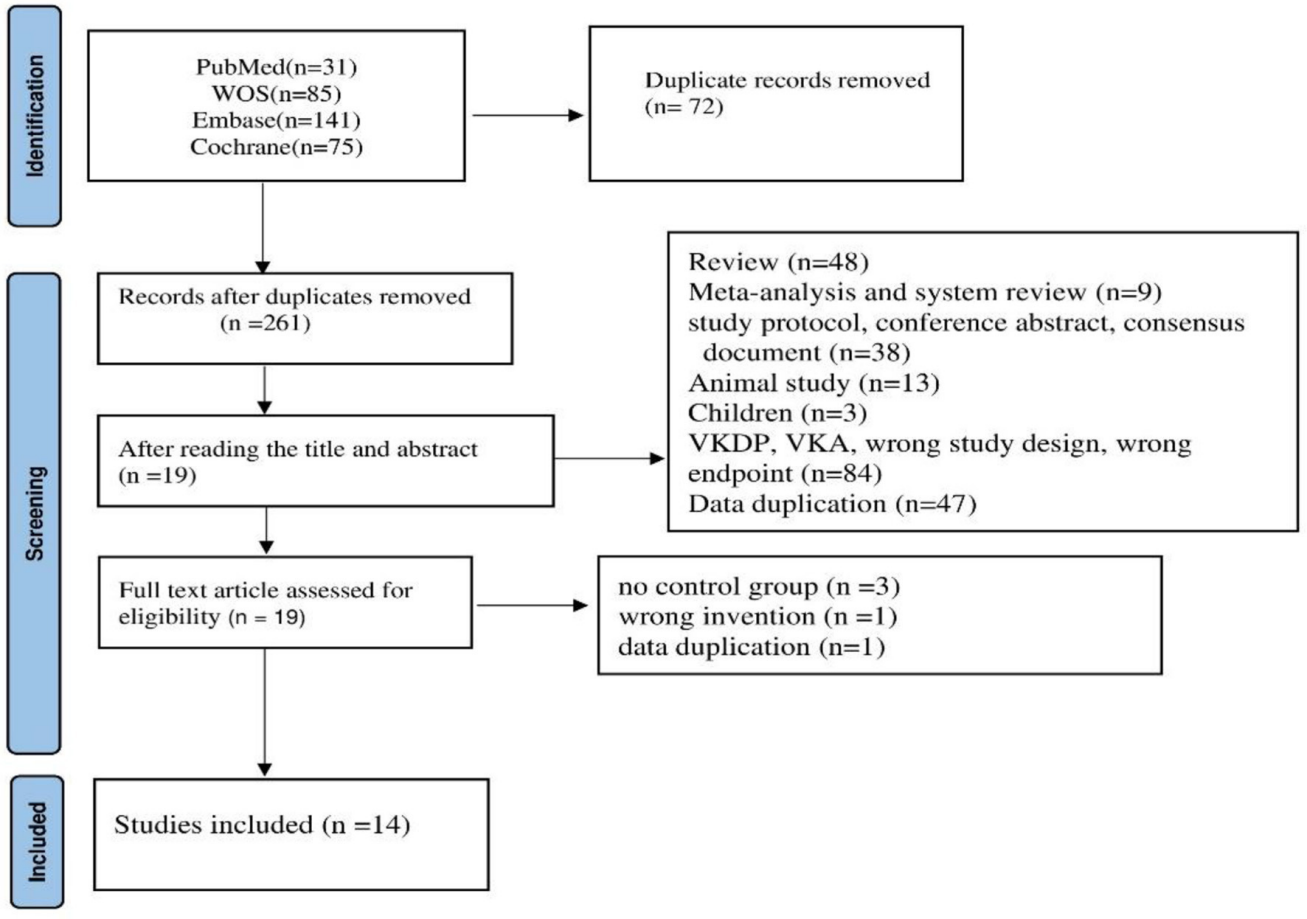
Flow chart of literature search process.

We conducted a comprehensive search across multiple databases and identified 332 articles. After removing duplicates, we excluded 242 studies based on title and abstract screening, leaving 19 studies for full-text review. After excluding three studies that lacked a control group, one article with duplicate data, and one study that used incorrect interventions, we included 14 RCTs in the final analysis. Figure 1 provides a summary of the included studies.

### Study characteristics

Our study included 14 RCTs with 1,842 participants initially enrolled. However, due to reasons, such as automatic withdrawal, adverse reactions, loss to follow-up, and other factors, the final number of participants included in the analysis was 1,533. Among them, four studies used VK1, nine studies used VK2 (MK-7), and one study only specified the use of VK without specifying the type of supplementation. Four of the included studies focused on type 2 diabetes mellitus (T2DM), while five studies focused on CKD patients (including both hemodialysis and non-hemodialysis patients). Two studies were conducted on postmenopausal women, one study focused on kidney transplant recipients (KTR), and the final two studies involved healthy individuals. Table 2 presents the main research characteristics.

Table 2Study characteristics.

**Table:** 

**References**	**Country**	**Center(s)**	**Population**	**Intervention**	**Control**	**Period of treatment (months)**	**Length of follow up (months)**
Witham et al. (20)	USA	2	CKD	400 μg MK-7, daily	Placebo	12	12
Levy-Schousboe et al. (21)	Denmark	4	T2DM	360 μg MK-7, daily	Placebo	24	24
Lees et al. (49)	UK	1	KTR	Menadiol diphosphate 5 mg, thrice weekly	Placebo	12	12
Bellinge et al. (12)	Australia	1	T2DM	Phylloquinone 10 mg, daily	Placebo	3	3
Zwakenberg et al. (28)	The Netherlands	1	D2M and CVD	360 μg MK-7, daily	Placebo	6	6
Shea et al. (13)	USA	1	Community	Multivitamin with 500 μg phylloquinone, daily	Multivitamin, daily	36	36
Bartstra et al. (29)	The Netherlands	3	T2DM	360 μg vitamin K2, daily	Placebo	6	6
De Vriese et al. (27)	Belgium	1	Hemodialysis, nonvalvular AF	2,000 μg MK-7, thrice weekly + rivaroxaban 10 mg, daily	Rivaroxaban 10 mg, daily/VKA	18	18
Knapen et al. (25)	The Netherlands	3	Postmenopausal women	180 μg MenaQ7, daily	Placebo	36	36
Holden et al. (39)	Canada	1	Hemodialysis	10 mg of phylloquinone, thrice weekly	Placebo	12	12
Oikonomaki et al. (26)	Greece	1	Hemodialysis	200 μg MK-7, daily	No treatment	12	12
Kurnatowska et al. (30)	The Netherlands	2	CKD3–5	90 μg MK-7 + 10 μg cholecalciferol, daily	10 μg cholecalciferol	9	9
Braam et al. (50)	The Netherlands	1	Postmenopausal women	1 mg VK 1 + 10 mg zinc, 150 mg magnesium + 8 μg VD	10 mg zinc, 150 mg magnesium, and 8 μg VD/placebo	36	36
Fulton et al. (31)	Scotland	1	Older, vascular disease	100 μg MK-7, daily	Placebo	6	6

**Table:** 

**References**	***N*** = **participation (analysis)**	**Intervention** ***N*** =	**Control** ***N*** =	**Age (SD) Intervention**	**Control**	**Female intervention**	**Control**	**Male intervention**	**Control**
Witham et al. (20)	159 (124)	80 (61)	79 (63)	67.3 ± 11	65.7 ± 13.5	32	30	47	57
Levy-Schousboe et al. (21)	48	24	24	62 ± 11	66 ± 11	5	6	19	18
Lees et al. (49)	90 (83)	45 (42)	45 (41)	56.3 ± 11.1	58.9 ± 7.8	13	14	32	31
Bellinge et al. (12)	154 (149)	76 (73)	78 (76)	65.2 ± 7.1	65.2 ± 7.1	27	26	51	50
Zwakenberg et al. (28)	68 (60)	35 (33)	33 (27)	69.1 ± 8.4	69.1 ± 8.4	9	7	26	26
Shea et al. (13)	388 (295)	200 (149)	188 (146)	68 ± 6	68 ± 5	59	62	141	126
Bartstra et al. (29)	68 (60)	35 (33)	33 (27)	69 ± 8	69 ± 8	9	7	26	23
De Vriese et al. (27)	132	42	46/44	79.6 ± 7.3	79.9 ± 7.04/80.3 ± 9.48	14	11/9	28	35/25
Knapen et al. (25)	244 (233)	120 (111)	124 (120)	59.8 ± 3.5	59.3 ± 3.1	120	124	0	0
Holden et al. (39)	86 (69)	41 (34)	44 (35)	63 ± 11.85	61 ± 16.3	14	23	27	21
Oikonomaki et al. (26)	102 (52)	44 (22)	58 (30)	70.18 ± 66.65	66.65 ± 16.4	NR	NR	NR	NR
Kurnatowska et al. (30)	42 (40)	29 (28)	13 (12)	NR	NR	NR	NR	NR	NR
Braam et al. (50)	181 (108)	63 (38)	58 (30)/60 (40)	55.4 ± 2.8	55.9 ± 2.8/54.1 ± 3.0	38	30/40	0	0
Fulton et al. (31)	80	40	40	76 ± 4.4	77.1 ± 4.8	19	17	21	23

Age, presented as mean ± SD; NR, no report; VKA, vitamin K antagonism; AF, atrial fibrillation.

### Risk of bias in studies

**Figure 2:**
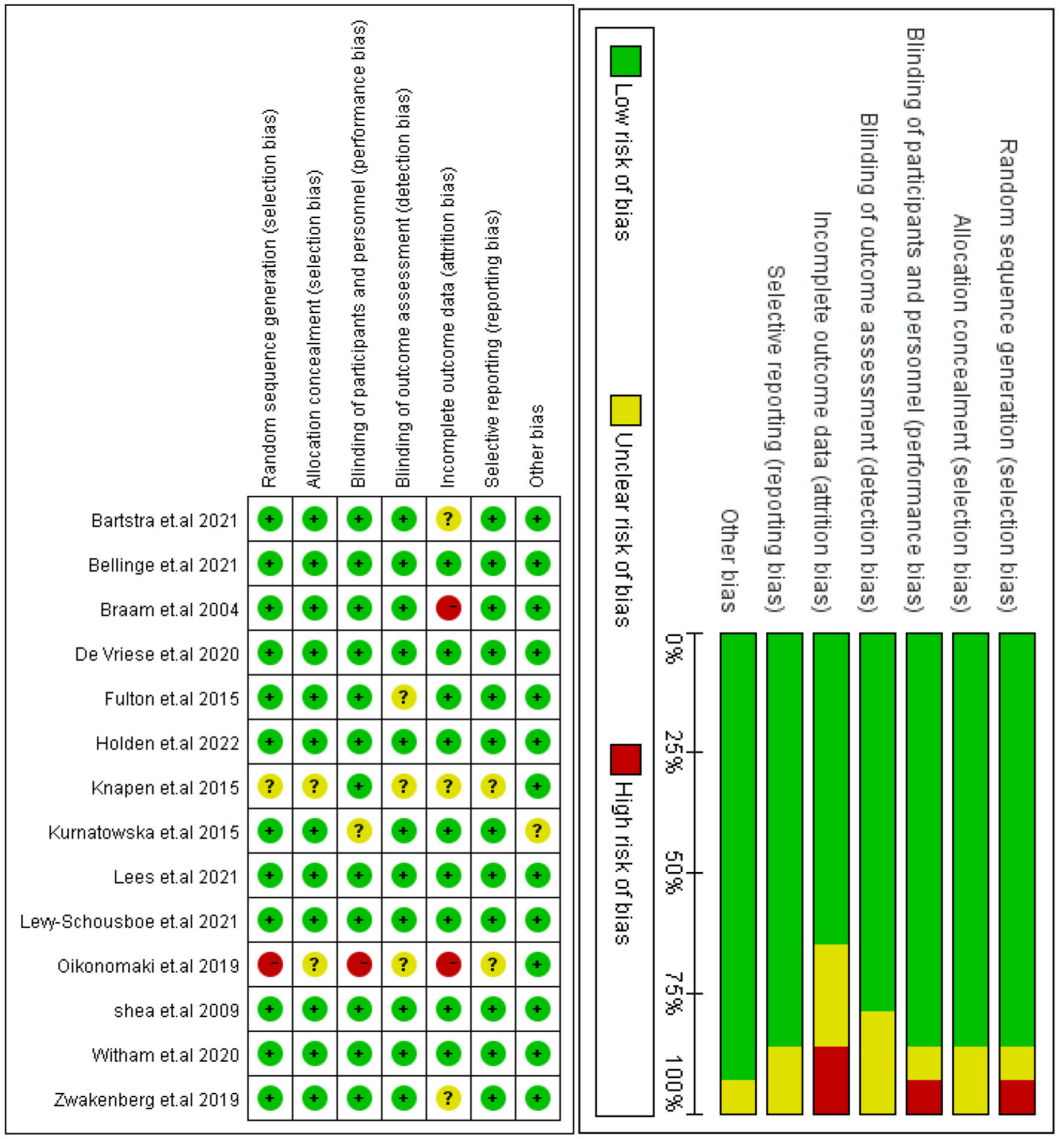
Risk of bias: review judgements about each risk of bias item for each included study.

Overall, seven studies presented a low risk of bias between all parameters. The research design methods in the Knapen et al. (25) study were poorly described. Sequence generation and allocation concealment were rated as ambiguous and highly biased in Oikonomaki et al.'s study (26). One trial used an open-label study design, which places it at high risk of bias in terms of participants and staff blinding (27). Additionally, it is unclear whether outcome data are completed for the study conducted by Zwakenberg et al. (28) with Bartstra et al. (29). Similarly, two studies made no mention of allocation concealment or blinding of outcome assessors for all outcomes (30, 31). Finally, Figure 2 presents the quality evaluation of the included RCTs.

### Study outcomes

#### VC

**Figure 3:**
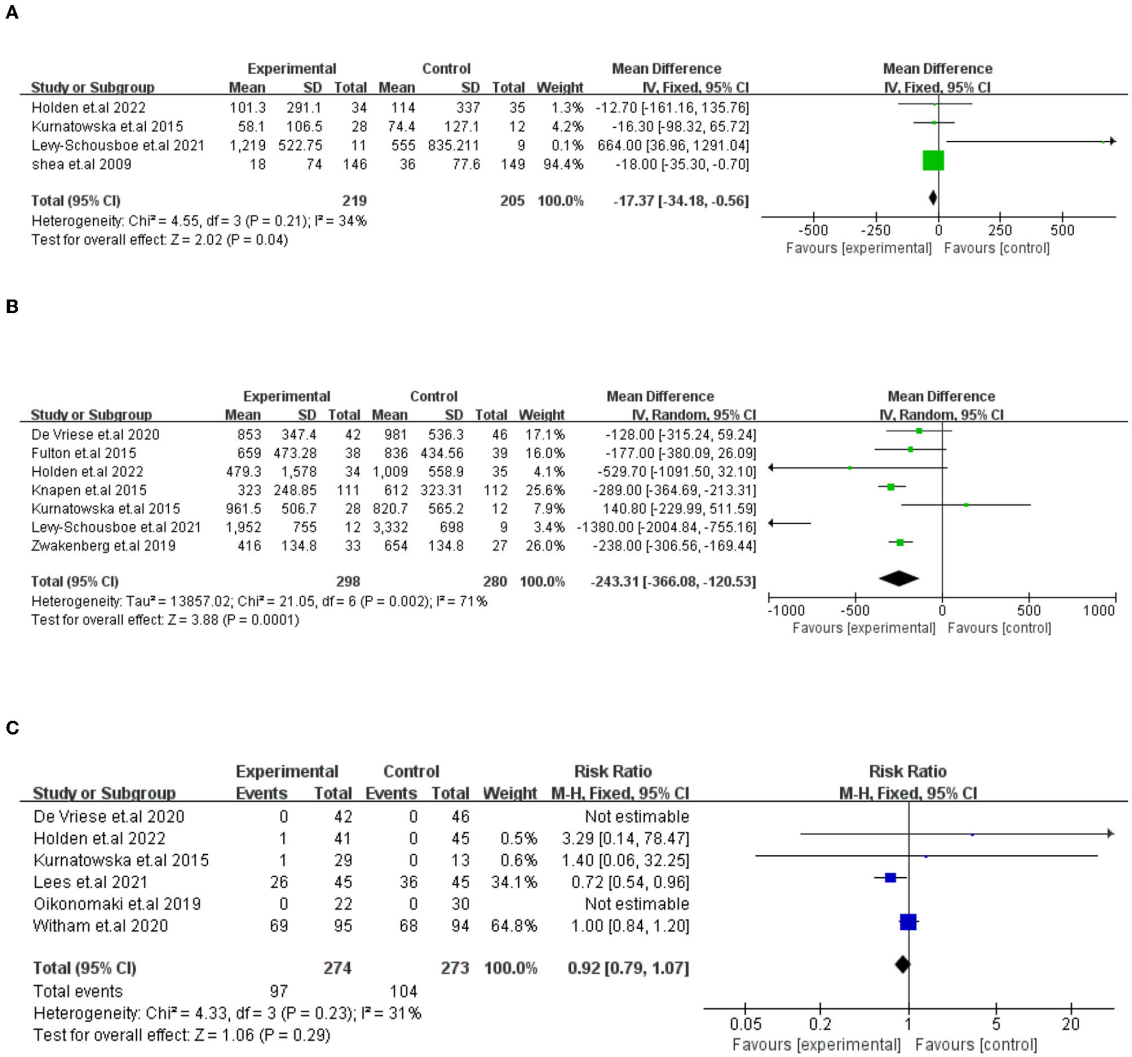
Forest plots showing the effect of VK intake on changes in CAC scores **(A)**, dp-ucMGP **(B)**, and advance events **(C)**. Data are presented as mean difference, risk radio and 95% CI.

① Vitamin K (VK) and VC of the change on CAC scores. A total of five studies compared VK supplementation on the change in CAC scores. One study (De Vriese et al.) was excluded because the results were expressed differently from other studies. In the end, four studies (424 participants) were included in the analysis. The results showed that VK supplementation has a significant effect on the decline in CAC scores, which indicated that VK supplementation slows the progression of CAC [*I*^2^ = 34%, MD = −17.37, 95% CI (−34.18, −0.56), *p* = 0.04; presented in Figure 3].
② Vitamin K (VK) and VC of other measures of artery calcification. A total of eight studies describing how VK supplementation affects the calcification of other arteries or valves are presented in Table 3. In Zwakenberg et al.'s (28) study, target-to-background ratios (TBRs) tended to rise in the MK-7 group compared with placebo (0.25,95% CI [−0.02, 0.51], *p* = 0.06), though it would not be statistically significant. Equally, in Bellinge et al.'s study, 10 mg VK1 daily supplementation also helps to reduce the occurrence of the development of newly calcifying lesions in the aorta (OR = 0.27, 95% CI: 0.08 to 0.94, *P* = 0.04), coronary arteries (OR = 0.35, 95% CI:0.16 to 0.78, *P* = 0.01), both coronary and aortic arteries (OR = 0.28, 95% CI: 0.13 to 0.63, *P* = 0.002) as detected using ^18^F-NaF PET. However, six other studies reported no significant effect of VK supplementation on vascular and valvular calcification.

**Table 3:** Measures of artery calcification.

**References**	**Outcome**	**Location of measurement**	**Within group intervention**	**Control**	**Difference between groups**	***p*-value**
Levy-Schousboe et al. (21)	Change in Agatston score (AU)	CVC, MD (95% CI)	416 (−213 to 1,045)	719 (226 to 1,212)	−303 (−1,117 to 512)	0.47
	Change in calcification volume	CVC, MD (95% CI)	346 (−165 to 857)	583 (194 to 973)	−237 (?890 to 416)	0.48
		CAC, MD (95% CI)	968 (134 to 1,802)	415 (−119 to 949)	553 (−445 to 1,550)	0.28
Lees et al. (49)	Distensibility (ascending/10^−3^ mmHg)	Aortic, MD (95% CI)	−0.3 (−0.6 to 0.1)	−0.1 (−0.4 to 0.3)	−0.23(−0.75 to 0.29)	0.377
	Descending (descending/10^−3^ mmHg)	Aortic, MD (95% CI)	−0.2 (−0.6 to 0.2)	−0.5 (−0.9 to −0.1)	0.23 (−0.32 to 0.78)	0.407
**References**	**Outcome**	**Location of measurement**	**Baseline intervention**	**Control**	**End of follow up intervention**	**Control**	**Difference between groups**	***p*** **-value**
Zwakenberg et al. (28)	Changes in TBR	Femoral arterial calcification, MD+SD	2.2 ± 0.7	2.1 ± 0.6	0.9 ± 0.55	−0.15 ± 0.44	0.25 (−0.02 to 0.51)	0.06
	Changes in calcification mass (CT)	Femoral arterial calcification, MD (IQR)	196.0 (32.5–424.0)	44.9 (9.6–409.5)	19.7 (2.2 to 51.4)	4.3(0.1 to 20.4)	0.5 (−0.23 to 1.36)	0.18
Oikonomaki et al. (26)	Agatston score (HU)	Aortic, MD + SD	7,827.88 ± 5,493.38	8,253 ± 6,298.94	10,412.53 ± 7,227.2	11,036.58 ± 9,053.34		NR
	Volume (mm 3)	Aortic, MD + SD	6,343.29 ± 4,176.29	6,529.25 ± 4,689.64	8,128.64 ± 5,534.46	8,609.25 ± 6,781.74		NR
	Mass (gr)	Aortic, MD + SD	2,394.42 ± 1,905.12	2,914.27 ± 3,786.69	3,009.51 ± 2,446. 57	3,557.06 ± 3,033.08		NR
**Reference**	**Outcome**	**Location of measurement**	**Within group intervention**	**Control**	**Univariate OR (95% CI)**	**Multivariate OR (95% CI)**	***p*** **-value**
De Vriese et al. (27)	Change of Agatston scores (%)	Total coronary artery, MD(IQR)	7,864 (4,135–14,019)	8,991 (4,165–22,185)	18.6% (7.2%−110.0%)	19.8% (7.2%−37.9%)	0.73
		Thoracic aorta, MD (IQR)	72 (8–489)	116 (22–346)	25.6% (8.2%−61.4%)	18.7% (4.6%−48.8%)	0.79
		Aortic & mitral valves, MD(IQR)	339 (40–1,028)	415 (71–2,276)	36.3% (3.1%−132.6%)	33.4% (5.8%−84.2%)	0.81
	Change of volume scores (%)	Total coronary artery, MD(IQR)	548 (108–993)	647 (195–1,199)	29.3% (9.7%−56.0%)	14.9% (2.7%−34.0%)	0.43
		Thoracic aorta, MD(IQR)	2,834 (1,478–4,541)	2,930 (1,352–6,244)	19.5% (7.3%−56.0%)	15.6% (5.1%−35.0%)	0.62
Bartstra et al. (29)	Change in arterial calcification mass score	Intracranial internal carotid artery, MD (IQR)	12 (3–25)	3 (0–35)	0 (−1; 5)	0 (−0; 2)	0.76
		Common carotid artery	3 (0–25)	2 (0–10)	0 (−0; 3)	0 (−1; 0)	0.20
		Coronary arteries	74 (13–165)	46 (1–148)	5 (−5; 12)	1 (−4; 11)	0.68
		Aorta	742 (322–1,337)	365 (39–1,144)	40 (−30; 125)	11 (0; 47)	0.55
		Iliac arteries	633 (242–1,148)	337 (66–764)	25 (6; 87)	5 (−4; 30)	0.07
		Leg arteries	309 (93–851)	90 (11–627)	35 (−8; 99)	7 (0; 47)	0.62
		Total arterial calcification	1,694 (812–3,584)	1,182 (235–2,445)	3 (−2; 16)	36 (1; 129)	0.38
Holden et al. (39)	Change of volume (mm^3^)	CAC, MD(IQR)	647.0 [302.0, 1,415.0]	326.5 [131.0, 957.0]	106.6 [32.0, 354.8]	95.0 [2.0, 381.0]	0.96
**Reference**	**Outcome**	**Location of measurement**	**Within group intervention**	**Control**	**Univariate OR (95% CI)**	**Multivariate OR (95% CI)**	***p*** **-value**
Bellinge et al. (12)	Development of ^18^F-NaF PET positive lesions	Coronary and aortic	26%	47.4%	0.39 (0.20, 0.78)	0.28 (0.13, 0.63)	**0.002**
		Coronary arteries	20.5%	38.2%	0.42 (0.20, 0.87)	0.35 (0.16, 0.78)	**0.01**
		Aortic	5%	17.1%	0.36 (0.12, 1.06)	0.27 (0.08, 0.94)	**0.04**

The meaning of the bold values refer to *p* < 0.05.

#### VK and dp-ucMGP

A total of seven trials (578 participants) compared VK supplementation, which showed a substantial impact on the decline in dp-ucMGP. The difference was statistically significant between the two groups [*I*^2^=71%, MD= −243.31, 95% CI (−366.08, −120.53), *p* = 0.0001; presented in Figure 3].

#### Adverse events

The prevalence and types of adverse events that occurred in study subjects were described in six trials (547 participants). In Witham et al.'s (20) study, adverse events accounted for a large proportion. Although no adverse events or effects were connected with VK2 supplementation after dialysis in De Vriese et al.'s study, 36 life-threatening or massive hemorrhage incidents took place in the 132 patients which need us to focus on the study of De Vriese et al. (27). The adverse events were not significantly different between the groups [*I*^2^ = 31%, RR = 0.92, 95% CI [−0.79, 1.07], *p* = 0.29; presented in Figure 3].

### Sensitivity analysis

**Figure 4:**
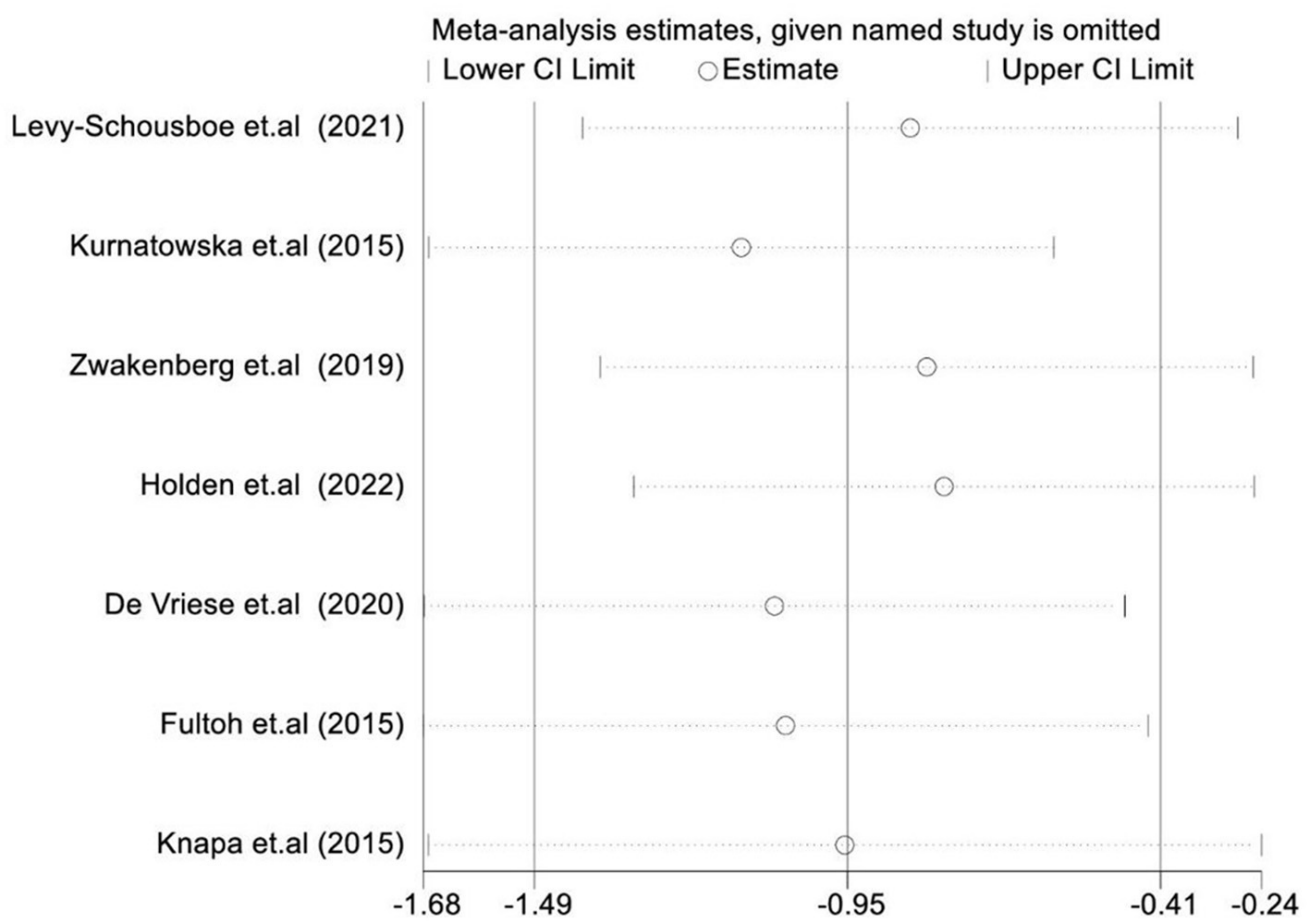
Sensitivity analyses.

We conducted a sensitivity analysis to investigate the effectiveness and safety of VK supplementation on VC and assess the stability and reliability of the meta-analysis results. After successively omitting each study, we found that the overall results had no significant impact, indicating that the conclusions drawn were stable and reliable. Figure 4 provides a summary of the results.

### Test for heterogeneity and report bias

**Figure 5:**
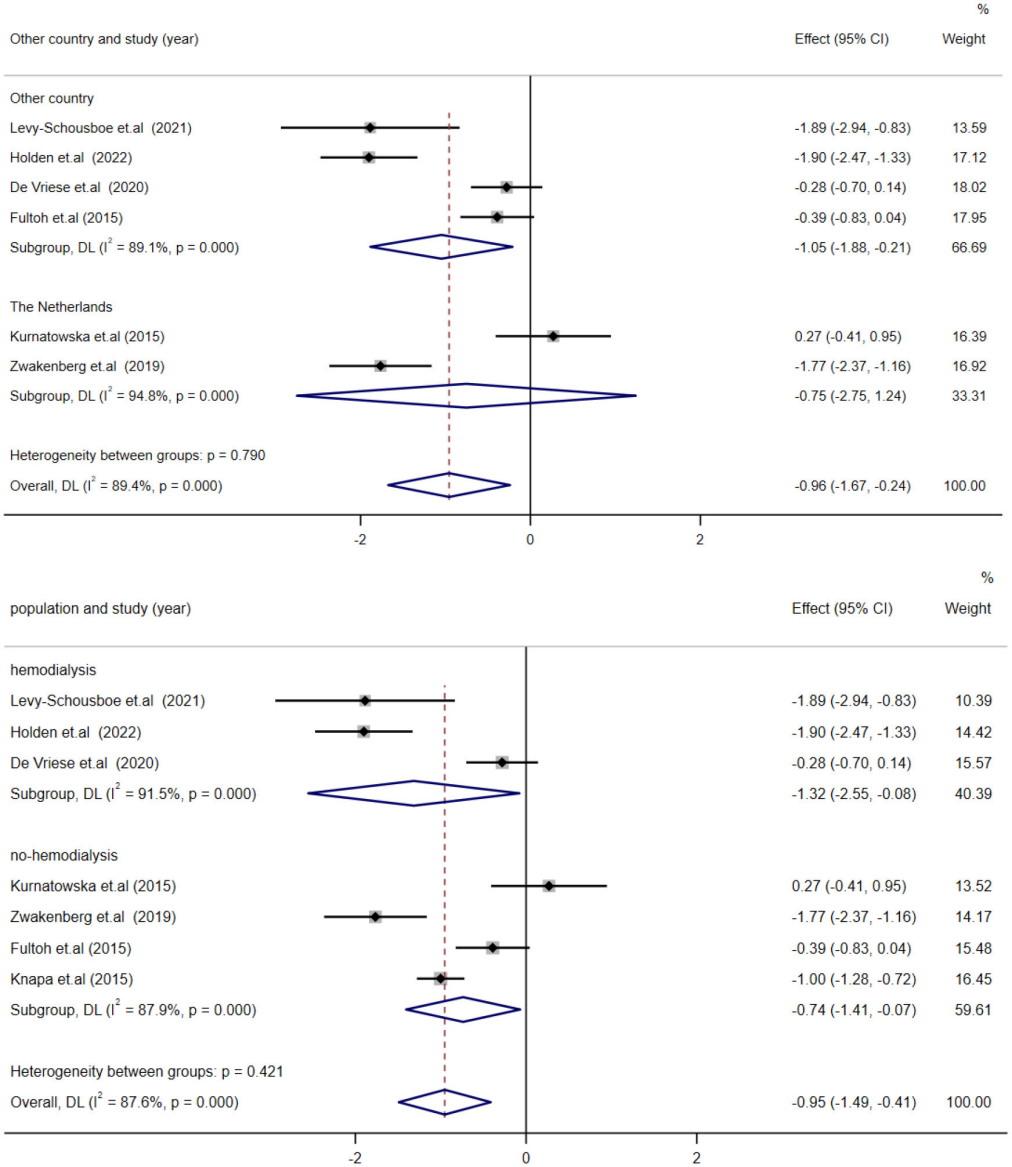
Subgroup analyses about country and population of dp-ucMGP.

Seven studies were compared for evaluating the impact of VK supplementation on dp-ucMGP. However, the results exhibited a high degree of heterogeneity (*I*^2^ = 71%). We performed a meta-regression with the MD in dp-ucMGP as the dependent variable and the sources of heterogeneity. Meta-regression revealed that there was no significant effect on VC of VK supplementation forms or doses, year, and duration of follow-up on univariate analysis (Table 4). There were insufficient observations on the country and population. Subgroup analyses were performed based on population (hemodialysis vs. no-hemodialysis) and country (the Netherlands vs. other countries) of dp-ucMGP. Similarly, a subgroup analysis showed that country and population are not the sources of heterogeneity (Figure 5). Due to the large differences in study outcomes and the small number of included articles, we did not test for publication bias.

**Table 4:** Meta-regression with the mean difference (%) in dp-uc MGP.

**Variable**	**Coefficient**	**95% CI**	***p*-value**	**Tau^2^**
Forms	−0.4123578	−1.509164 to 0.6844482	0.378	0.4862
Year	−1.901139	−4.68688 to 0.8846013	0.099	0.3276
Duration of follow-up	−1.00118	−5.289889 to 3.287529	0.421	0.9593
Dose	−0.7596538	−1.780453 to 0.2611456	0.114	0.6445

## Discussion

We performed a systematic review and meta-analysis of the effect of VK supplementation on the progression of VC. Current research indicates that VK supplementation mitigates VC, especially CAC. Additionally, VK supplementation improves VK status, based on the carboxylation status of dp-ucMGP. Moreover, few adverse events were reported. These findings are consistent with those of a meta-analysis published in 2018 and have been confirmed by Kosciuszek et al. (32, 33). However, Vlasschaert et al. (34) stated that while VK supplementation may increase the carboxylation of dp-ucMGP, its role in preventing VC is uncertain based on the available data.

### VK and calcification of vessels or valves

In this article, we testify that VK supplementation slows the development of CAC. Belling et al. found that 3 months of VK1 supplementation decreased the development of newly calcifying lesions in the aorta and the coronary arteries, as detected by using ^18^F-NaF PET (12). Similarly, the study by Shea et al. put forward that VK supplementation delayed the course of pre-existing CAC in healthy women and elderly men (13). However, eight other trials that measured major arterial or valve calcification reported no appreciable benefits of VK intake. The role of VK in CAC seems to remain controversial.

Coronary artery calcification (CAC) means the existence of coronary artery disease that is accompanied by the progression of advanced atherosclerosis and is a well-established predictive indicator of future CVD events (35). It is easily identified by radiography as well as by CT and intravascular imaging (35, 36). At the same time, the CT scan-based Agatston score can serve as a quantitative measure of CAC (37). Their application has been expanded to examine other blood vessels. However, the Agatston score fails to capture information about the regional distribution of calcified plaque, and large variability tends to occur in baseline differences of CAC scores, which contribute to the negative results (38, 39). As a non-invasive and quantitative imaging technique, ^18^F-NaF PET provides a biomarker of calcification activity and detects new calcium generation outside the resolution of CT (40). In Zwakenberg et al.'s (28) study, ^18^F-NaF activity tended to rise in the MK-7 group compared with the placebo group, which is contrasting with what we found. The discrepancy in the baseline calcification mass and different calcification areas detected by ^18^F-NaF uptake may have played a role in the disappointing trial outcome (41). In addition, a related study evaluated the uptake of ^18^F-NaF PET in relation to gender and the number of cardiovascular risk factors (42). In the recent presentation, invasive imaging techniques, such as angiography, with or without optical coherence tomography, provide a higher spatial resolution of mineralized sites inside and along the artery walls (43). Although these are more sensitive for the identification of VC, their applicability is restricted as an invasive procedure. Overall, limitations in image resolution and other factors may bias the evaluation of calcification scores when using traditional imaging modalities such as CT scans. However, despite these limitations, we have found conclusive evidence that VK intake may impede VC, particularly CAC. Another way to assess the status of VK is by measuring dp-ucMGP levels in the blood, which has been linked to surrogated biomarkers of VC, vascular stiffness, and cardiovascular outcomes (44).

### VK and dp-ucMGP

Matrix Gla proteins (MGP) are one of the most effective naturally occurring inhibitors of VC and require VK as a cofactor to become bioactive through post-translational γ-carboxylation and phosphorylation (45). The functional status of VK related to specific tissues can be reflected indirectly by measuring un-carboxylation or carboxylation MGP *in vivo*. However, other types of MGP (except for dp-ucMGP) have a high affinity for precipitated calcium salts and hydroxyapatite, preventing them from freely entering the bloodstream (46). In addition, determining VK content in the plasma is challenging due to the low circulating level of VK, the non-polar nature of VK, and the interference from lipids (5). As a result, measuring dp-ucMGP levels in the blood could be a useful alternative way to assess the status of VK, which is associated with a surrogate biomarker of vascular health (44).

A total of seven trials (578 participants) were compared, and there was a considerable impact on the decline in dp-ucMGP after VK supplementation. Holden et al. (39) conducted parallel RCTs in hemodialysis patients in 2022, which indicated that dp-ucMGP levels declined by 86% in the VK1 group. Levy-Schousboe et al.'s study showed that despite continuing VK supplementation within hemodialysis patients, dp-ucMGP decreased from the baseline in year 1 and increased again in year 2. It seems to demonstrate that VK supplementation significantly decreases dp-ucMGP levels but cannot prevent a subsequent increase (21). Moreover, another study of hemodialysis patients revealed that lengthy VK2 intake at therapeutic doses and VK antagonism withdrawals do not normalize systemic dp-ucMGP levels, but rather just reduce them (27). The results of the three studies are highly consistent, but they also raise questions worth considering. Specifically, the findings prompt us to inquire why VK supplementation does not restore dp-ucMGP to normal levels in hemodialysis patients.

There are two reasons to explain why dp-ucMGP levels cannot be normalized in hemodialysis patients after VK supplementation. On the one hand, multiple studies have confirmed that dp-ucMGP levels are significantly elevated in hemodialysis patients, indicating VK deficiency. The causes of VK insufficiency in hemodialysis are multifaceted and include inadequate intake, uremic suppression of the VK cycle, and possibly interfering with VK absorption by phosphate binders. On the other hand, dp-ucMGP concentrations are decided by VK status and total MGP levels which have been shown to increase with age and in CVD (47, 48). Furthermore, changes in dp-ucMGP levels were not only seen in hemodialysis patients but also in other patients. Fulton et al., Knapen et al., and Kurnatowska et al. demonstrated that dp-ucMGP levels fell significantly after VK supplementation in other patients. The result is consistent with Zwakenberg et al.'s study. Numerous studies focused on dp-ucMGP alterations in reaction to VK therapy, and it has not been mentioned whether decreasing the level of dp-ucMGP after VK supplementation could well inhibit or alter the advancement of VC. However, we have confirmed that VK supplementation reduces the absolute level of dp-ucMGP.

## Conclusion and recommendation

As research progresses, numerous studies have explored ways to inhibit VC, but the complexity and variety of its pathophysiology have presented obstacles. The majority of trials are typically small, single-center studies, and are varied in terms of the type of VK administered, dose of VK, participants studied, results measured, and duration of follow-up. However, with the emergence of more RCTs in the past 2 years, these studies have demonstrated higher participant retention rates, larger cohorts, and longer follow-up periods. In addition, more studies are conducted particularly in the high-risk group of people with DM, CAC, and CKD, who would most potentially benefit from the therapeutic intervention being tested. Furthermore, new technologies, such as ^18^F-NaF PET, have emerged to identify early and active areas of calcification with greater sensitivity than CT. In spite of this, few researchers have found evidence of VK supplementation plays a significant role in inhibiting the progression of VC. A limitation of our study is the notable variability in the results of the included research. Nonetheless, we contribute to addressing an important and unsolved issue by providing evidence that supports the potential of VK supplementation in mitigating VC, especially in the case of CAC. VK deficiency is common in populations who are at high risk of CVD and CKD and may be more easily treated with VK supplementation than with a change of lifestyle. In addition, VK supplementation does not produce serious adverse effects and may be advantageous as a long-term strategy to enhance vascular health and decrease CVD risk. In the future, we encourage further RCTs to confirm the efficacy of VK therapy. Before this can be translated into clinical practice, more studies are required.

## Data availability statement

Publicly available datasets were analyzed in this study. This data can be found here: All data in this paper were collected from databases, including PubMed, the Cochrane Library, Embase databases, and Web of science until August 2022.

## Author contributions

TL and WPT conceptualized the idea and created the protocol for this study. TL and YW developed the search strategy and performed the data collection. TL analyzed the data and drafted the manuscript. All authors have read and approved the final manuscript.
