# Supplementary material for: Vitamin K supplementation and vascular calcification: a systematic review and meta-analysis of randomized controlled trials
Source: Front Nutr. 2023 May 12;10:1115069. doi: 10.3389/fnut.2023.1115069 (PMC10218696; doi:10.3389/fnut.2023.1115069)
Supplement: Supplementary file 1 [file Data_Sheet_1.docx]

检索策略

Comment:

ID Search Hits

#1 (vitamin k):ti,ab,kw (Word variations have been searched) 2774

#2 MeSH descriptor: [Vitamin K] explode all trees 669

#3 #1 or #2 2774

#4 (Aquamephyton or Konakion or Phyllohydroquinone or Phytomenadione or Vitamin K1 or Phytonadione or Phylloquinone):ti,ab,kw (Word variations have been searched) 353

#5 MeSH descriptor: [Vitamin K 1] explode all trees 138

#6 #4 or #5 369

#7 (Menaquinones or Vitamin K2 or Menaquinone or Vitamin K Quinone):ti,ab,kw (Word variations have been searched) 307

#8 MeSH descriptor: [Vitamin K 2] explode all trees 150

#9 #7 or #8 326

#10 (Vascular Calcinosis or Calcinosis, Vascular or Calcinoses, Vascular or Calcification, Vascular or Vascular Calcifications or Calcifications, Vascular or Vascular Calcinoses):ti,ab,kw (Word variations have been searched) 793

#11 MeSH descriptor: [Vascular Calcification] explode all trees 186

#12 #10 or #11 794

#13 (Calcinosis, Tumoral or Tumoral Calcinoses or Calcinoses, Tumoral or Tumoral Calcinosis or Calcinoses or Pathologic Calcification or Calcification, Pathologic or Microcalcification or Microcalcifications or Microcalcinosis or Microcalcinoses):ti,ab,kw (Word variations have been searched) 414

#14 MeSH descriptor: [Calcinosis] explode all trees 562

#15 #13 or #14 853

#16 (Randomized Controlled Trial or random*):ti,ab,kw (Word variations have been searched) 1167872

#17 MeSH descriptor: [Randomized Controlled Trial] explode all trees 118

#18 #16 or #17 1167872

#19 #3 or #6 or #9 2930

#20 #12 or #15 1348

#21 #18 and #19 and #20 75
